# Comparative analysis of latex transcriptomes reveals the potential mechanisms underlying rubber molecular weight variations between the *Hevea brasiliensis* clones RRIM600 and Reyan7-33–97

**DOI:** 10.1186/s12870-021-03022-5

**Published:** 2021-05-29

**Authors:** Shichao Xin, Yuwei Hua, Ji Li, Xuemei Dai, Xianfeng Yang, Jinu Udayabhanu, Huasun Huang, Tiandai Huang

**Affiliations:** Key Laboratory of Biology and Genetic Resources of Rubber Tree, Ministry of Agriculture and Rural Affairs; State Key Laboratory Incubation Base for Cultivation & Physiology of Tropical Crops, Rubber Research Institute, Chinese Academy of Tropical Agricultural Sciences, Haikou, 571101 P. R. China

**Keywords:** Natural rubber, *Hevea brasiliensis*, Rubber molecular weight, Latex transcriptome, Rubber particle size

## Abstract

**Background:**

The processabilities and mechanical properties of natural rubber depend greatly on its molecular weight (MW) and molecular weight distribution (MWD). However, the mechanisms underlying the regulation of molecular weight during rubber biosynthesis remain unclear.

**Results:**

In the present study, we determined the MW and particle size of latex from 1-year-old virgin trees and 30-year-old regularly tapped trees of the *Hevea* clones Reyan7-33–97 and RRIM600. The results showed that both the MW and the particle size of latex varied between these two clones and increased with tree age. Latex from RRIM600 trees had a smaller average particle size than that from Reyan7-33–97 trees of the same age. In 1-year-old trees, the Reyan7-33–97 latex displayed a slightly higher MW than that of RRIM600, whereas in 30-year-old trees, the RRIM600 latex had a significantly higher MW than the Reyan7-33–97 latex. Comparative analysis of the transcriptome profiles indicated that the average rubber particle size is negatively correlated with the expression levels of rubber particle associated proteins, and that the high-MW traits of latex are closely correlated with the enhanced expression of isopentenyl pyrophosphate (IPP) monomer-generating pathway genes and downstream allylic diphosphate (APP) initiator-consuming non-rubber pathways. By bioinformatics analysis, we further identified a group of transcription factors that potentially regulate the biosynthesis of IPP.

**Conclusions:**

Altogether, our results revealed the potential regulatory mechanisms involving gene expression variations in IPP-generating pathways and the non-rubber isoprenoid pathways, which affect the ratios and contents of IPP and APP initiators, resulting in significant rubber MW variations among same-aged trees of the *Hevea* clones Reyan7-33–97 and RRIM600. Our findings provide a better understanding of rubber biosynthesis and lay the foundation for genetic improvement of rubber quality in *H. brasiliensis*.

**Supplementary Information:**

The online version contains supplementary material available at 10.1186/s12870-021-03022-5.

## Background

Natural rubber (NR) is an indispensable raw material in the manufacture of many thousands of industrial products. The Brazilian rubber tree (*Hevea brasiliensis*) is currently the only established commercial source of NR due to its high productivity of rubber with excellent physical properties [1]. In *Hevea* tree, NR molecules are biosynthesized using isopentenyl pyrophosphate (IPP) as a monomeric subunit, which is mainly synthesized by the cytosolic mevalonate pathway from acetyl-CoA [2, 3]. In the cytosol, IPP is initially converted by IPP-isomerase to its isomer, dimethylallyl diphosphate (DMAPP), which is subsequently condensed with IPP in the *trans*-configuration to form geranyl pyrophosphate (GPP), farnesyl pyrophosphate (FPP) and geranylgeranyl pyrophosphate (GGPP) [4]. With these *trans*-short-chain prenyl pyrophosphates serving as allylic primer substrates, the rubber molecules are biosynthesized by sequential condensation of IPP in *cis*-configuration via a carbocationic reaction [3, 5]. The enzyme rubber transferase (RT-ase) catalyses such a reaction at the surface of specialized organelle-like structures surrounded by a monolayer membrane in the cytosol of laticifer cells, which are known as rubber particles (RP), and the resulting hydrophobic rubber chains are compartmentalized in the interior of these globules [6–8]. Notably, in *Hevea* trees, adjacent laticifer cells anastomose to form an extensive network of laticiferous vessels within the bark tissue, in which the rubber particles repel each other to avoid fusion in an aqueous suspension known as latex [9, 10]. As a result, the raw polymer can be harvested from the *Hevea* tree by making an incision in the bark and collecting the latex freely flowing out of the vessels [5]. In this manner, a large amount of NR is produced in an efficient, sustainable and environmentally friendly manner for large-scale commercial use.

The MW and molecular weight distribution (MWD) of natural rubbers have large impacts on the processabilities and mechanical properties of rubber products [1]. NR with a higher MW showed better mechanical properties, such as greater tensile strength, tear strength resilience and abrasion resistance [11, 12]. NR with a broader MWD was found to have a faster carbon black incorporation time, i.e., better processability, than those with a narrower MWD [13, 14]. In in vitro rubber synthesis assays, the rates of both rubber molecule initiation and polymerization and the final polymer molecular weight depended greatly upon substrate concentrations, the ratio of IPP and initial allylic molecules, and the intrinsic properties of the rubber transferases [7, 15]. A number of metal ions have also been reported to be necessary cofactors for binding substrates to active sites, thereby regulating the initiation rate, biosynthetic rate and molecular weight of rubbers produced in vitro [16]. Thus, Cherian et al. [3] suggest that the laticifer environment may play a significant role in molecular weight regulation. Additionally, the size of rubber particles is considered an important parameter affecting latex molecular weight in *Hevea* trees. Small rubber particles are composed of higher MW rubbers and have essentially higher rubber transferase activity than larger rubber particles [17–20]. And the distinctly distributed rubber particle proteins between these two types of rubber particles, such as small rubber particle protein (SRPP) and rubber elongation factor (REF), may be involved in the regulation of polymer chain elongation and mature rubber molecular mass [21, 22]. It is also worth noting that the MW and MWD of rubbers are closely correlated with *Hevea* clone and age. Older rubber trees showed an increase in the MW of rubber [23], and fresh latex from multiple clones growing in the same rubber trial showed great variations in MW and MWD [24, 25]. These experimental findings suggest that rubber MW is controlled by multiple factors, including laticifer environments, rubber particle types, genetics and developmental stages.

With the current advances in next-generation sequencing technology, several genomes, transcriptomes and proteomes for a number of *H. brasiliensis* clones and other rubber-producing species have been reported [26–31]. However, these studies focused mainly on the identification of key genes involved in processes such as rubber biosynthesis, latex flow, stress tolerance, laticifer cell development and tapping panel dryness (TPD). Less attention was given to clarifying the mechanisms underlying rubber molecular weight regulation. Based on the above-mentioned clonal and age-related differences in *Hevea* rubbers, it would be worthwhile to comparatively analyze the MW and RP size together with the transcriptome profiles of latex from different *Hevea* clones at different ages and link the MW and MWD variations with gene expression, which may provide an opportunity to understand the regulatory mechanisms of molecular weight during rubber biosynthesis.

RRIM600 is a *Hevea* clone originating from a cross between TJIR1 × PB86 in Malaysia and is one of the most widely planted rubber clones worldwide [32]. RRIM600 is an average latex-yielding clone with a production of ~ 1350 kg of latex per hectare per year, while its hybrid offspring Reyan7-33–97 is a high-yielding clone (~ 1959 kg/ha/year) with strong resistance to wind damage and low temperature, which makes it the most popular rubber tree clone in China [2, 33, 34]. In our present study, we investigated the MW, rubber particle size and distributions of latex from 1-year-old virgin trees and 30-year-old regularly tapped trees of the clones Reyan7-33–97 and RRIM600. By comparative analysis of latex transcriptomes, we studied the expression of rubber biosynthesis-related pathways, rubber particle-associated proteins, and transcription factors that are potentially involved in the formation of clonal differences in latex. Our results enriched publicly available *Hevea* transcriptomic resources and provided insights into molecular weight regulatory mechanisms during natural rubber biosynthesis, and may also accelerate the genetic improvement of rubber trees to produce high quality rubbers.

## Results

### Comparative analysis of latex MW and rubber particle size

**Fig. 1:**
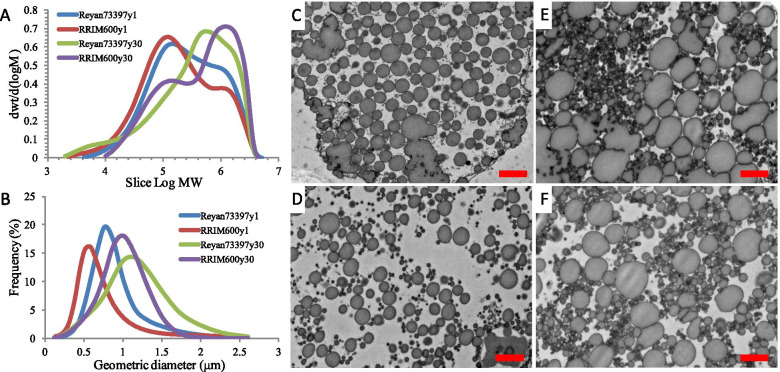
Comparative analyses of the molecular weight and rubber particle size of latex from 1-year virgin trees and 30-year-old regularly tapped trees of Reyan7-33–97 and RRIM600 clones. **a** The molecular weight distributions of four latex samples. **b** The particle size distributions of four latex samples. **c** to **f** indicate the TEM images of rubber particles in the laticifers of Reyan73397y1, RRIM600y1, Reyan73397y30 and RRIM600y30 trees, respectively. Scale bars represent 1 μm

The samples collected from 1-year-old virgin trees and 30-year-old regularly tapped trees of the clones Reyan7-33–97 and RRIM600 were designated Reyan73397y1, RRIM600y1, Reyan73397y30 and RRIM600y30, respectively. The number-average molecular weight (MN), MW and MWD (calculated as MW/MN) of the latex samples were determined by GPC, as shown in Table 1 and Fig. 1. As the age of *Hevea* trees increased from 1 to 30 years, the MW of Reyan7-33–97 latex increased from 4.77 × 10^5^ to 6.51 × 10^5^, and the MWD also increased from 5.53 to 7.19, while the latex from RRIM600 trees showed that the MW and MN dramatically increase from 4.26 × 10^5^ to 8.28 × 10^5^ and 7.18 × 10^4^ to 17.73 × 10^4^, respectively, with a slight decrease in MWD (from 5.94 to 4.67). In trees of the same age, the Reyan73397y1 latex displayed a slightly higher MW than that of RRIM600y1, whereas the RRIM600y30 latex had a significantly higher MW than that of Reyan73397y30 in the same trial (Table 1). As shown in Fig. 1a, these latex samples have a MW range from 2.0 × 10^3^ to 5.0 × 10^6^ and comprise a high molecular weight fraction (> 10^6^) and a low molecular weight fraction (< 10^5^). Latex from Reyan7-33–97 trees aged 1 and 30 years showed a skewed unimodal MW distribution with a shoulder in the high molecular weight region, while the latex obtained from RRIM600 trees showed a typical bimodal distribution (Fig. 1a). With increasing tree age, the latex from both Reyan7-33–97 and RRIM600 trees showed a dramatic increase in the high molecular weight fraction. In 1-year-old trees, latex from Reyan73397y1 had more high MW rubbers than that from RRIM600y1. In 30-year-old trees, the latex of Reyan73397y30 comprises more low MW rubbers (< 2.0 × 10^4^), and the position of the high MW peak is smaller than that of RRIM600y30 latex (Fig. 1a).

**Table 1:** The number-average molecular weight (MN), weight-average molecular weight (MW) and rubber particle size of latex from 1-year virgin trees and 30-year-old regularly tapped trees of Reyan7-33–97 and RRIM600 clones

Latex Samples	MN (× 10^4^)	MW (× 10^5^)	MWD (MW/MN)	Geometric Mean Diameter (μm)
Reyan73397y1	8.62 ± 1.84	4.77 ± 0.65	5.53	0.72
RRIM600y1	7.18 ± 0.39	4.26 ± 0.22	5.94	0.69
Reyan73397y30	9.05 ± 2.16	6.51 ± 0.88	7.19	0.85
RRIM600y30	17.73 ± 4.78	8.28 ± 0.58	4.67	0.79

Fresh latex samples were analyzed by laser light scattering (LLS) to determine the particle size, a factor potentially affecting the MW of NR (Table 1 and Fig. 1b). The results showed that the geometric mean diameter of RPs varies with age and clone. Latex from 1-year-old trees showed a particle size distribution of predominately less than 1 μm, while those of 30-year-old trees contained particles having a larger size (predominately in between 0.5 μm and 2 μm). On average, RP sizes are smaller in latex from RRIM600 trees than in Reyan7-33–97 trees of the same age. Transmission electron microscopy (TEM) images of ultrathin cross sections showed that the rubber particles within bark laticifers are predominantly spherical in shape. As shown in Figs. 1 c-f, the size of the RPs correlates well with the LLS measurements: the latex from 30-year-old trees has larger RPs than that from 1-year-old trees, and the RPs in RRIM600 latex tend to be smaller than those in Reyan7-33–97 latex. In both laticifers of Reyan7-33–97 at ages 1 and 30, we observed that some elongated particles appeared to result from aggregation, while in the analyzed sections of RRIM600 at ages 1 and 30, the rubber particles are uniformly dispersed.

### Transcriptome sequencing and analysis of differentially expressed genes (DEGs)

**Fig. 2:**
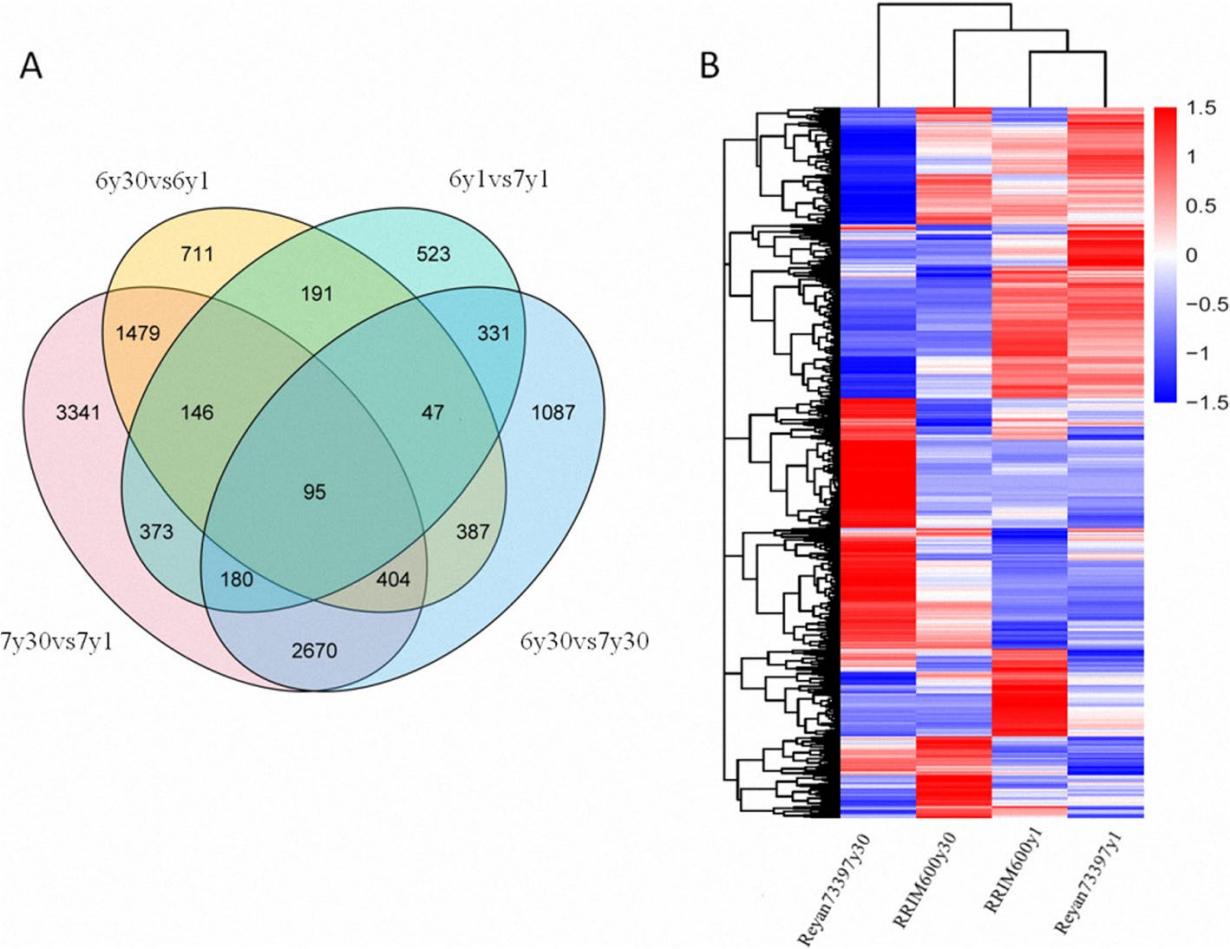
Analysis of differentially expressed genes among four latex samples. **a** Venn diagram showing the numbers of overlapping and uniquely differentially expressed genes in pairwise comparisons of RRIM600y1 (6y1), Reyan73397y1 (7y1), RRIM600y30 (6y30) and Reyan73397y30 (7y30). **b** Hierarchical clustering of differentially expressed genes in four latex samples

To understand the transcriptional variations underlying the clonal difference in rubber MW, we sequenced and analyzed the transcriptome files of these latex samples. Total RNA was isolated and sequenced using the Illumina paired-end sequencing approach. The adaptors, low-quality reads and contaminated reads were filtered out, and an average number of 6.45 Gb, 6.60 Gb, 6.35 Gb, and 6.39 Gb clean bases were generated for Reyan73397y1, RRIM600y1, Reyan73397y30 and RRIM600y30, respectively. These clean reads were then mapped to the *Hevea* reference genome, with mapping rates ranging from 95.53%—96.51% (Table S1). The gene expression patterns for each latex sample were calculated as fragments per kilobase of transcript per million fragments mapped (FPKM). A total of 34,907 genes with detectable expression levels were obtained from four latex samples. To analyze the sequencing results, the comparisons of RRIM600y1 vs. Reyan73397y1 (6y1vs7y1) and RRIM600y30 vs. Reyan73397y30 (6y30vs7y30) were performed to investigate the transcriptional regulation underlying the clonal MW difference, while RRIM600y30 (6y30) and Reyan73397y30 (7y30) were respectively compared with RRIM600y1 (6y1) and Reyan73397y1 (7y1) to study age-related latex variation. The numbers of DEGs for these four comparisons are shown in Table 2. A total of 2648 genes were upregulated and 2553 genes were downregulated in the latex of RRIM600y30 compared with that of Reyan73397y30. Interestingly, the comparison 7y30vs7y1 showed the most DEGs, at 8688, indicating distinct transcriptional regulation in Reyan7-33–97 latex as tree age increased. A Venn diagram was constructed to show the relationships of DEGs among different comparisons. As shown in Fig. 2a, 2124 DEGs overlapped among the comparisons 6y30vs6y1 and 7y30vs7y1, suggesting their roles in age-related differences in latex samples. The 653 overlapping DEGs between comparisons 6y1vs7y1 and 6y30vs7y30 are supposed to have clonally different expression patterns. Hierarchical cluster analysis of gene expression profiles indicated that DEGs obtained from the transcriptome sequencing analysis could be divided into 4 clusters, and the genes in the same subcluster had similar expression patterns among these latex samples (Fig. 2b and Fig. S1). The expression patterns of DEGs in Reyan73397y30 were less consistent with those in RRIM600y30, RRIM600y1 and Reyan73397y1 (Fig. 2b). To validate the results obtained from the transcriptome analyses, the expression levels of 17 key rubber biosynthesis genes and regulators were further studied by qRT-PCR. As shown in Fig. S2, the qRT-PCR results were significantly correlated with those of RNA-seq (R^2^ = 0.950), indicating that the expression data obtained by transcriptome sequencing were reliable.

**Table 2:** Numbers of the DEGs across four comparisons

	6y1vs7y1	6y30vs7y30	6y30vs6y1	7y30vs7y1
Upregulated	840	2648	1302	3797
Downregulated	1046	2553	2158	4891
Total	1886	5201	3460	8688

### Expression levels of genes related to rubber biosynthesis

**Fig. 3:**
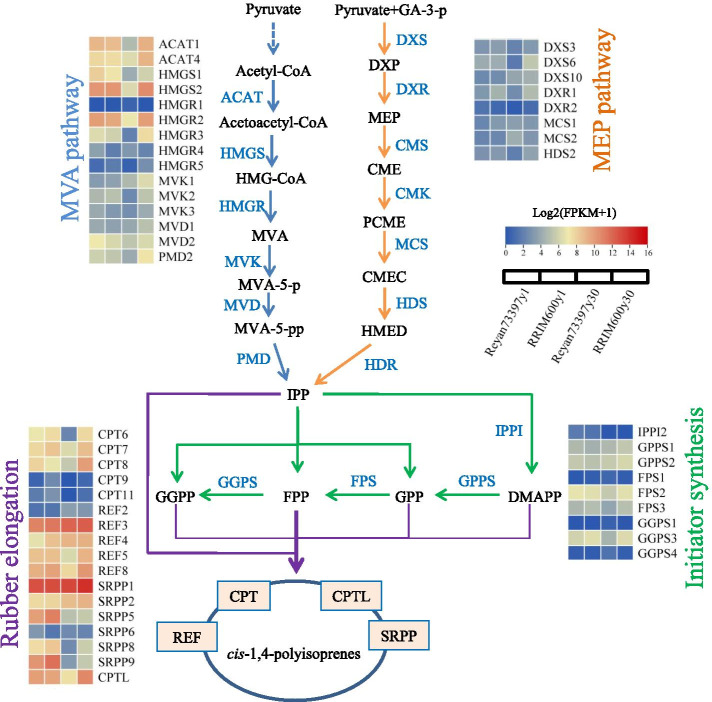
Heatmap of FPKM values (Log2(FPKM + 1)) for differentially expressed rubber biosynthesis genes. The lines represent duplicate genes and the columns represent the latex samples Reyan73397y1, RRIM600y1, Reyan73397y30 and RRIM600y30 from left to right. Abbreviations: ACAT, acetyl-CoA acetyltransferase; HMGS, hydroxymethyglutaryl coenzyme A synthase; HMG-CoA, hydroxymethylglutaryl coenzyme A; HMGR, hydroxymethyglutaryl coenzyme A reductase; MVA, Mevalonate; MVK, mevalonate kinase; MVA-5-p, mevalonate-5-phosphate; MVD, phosphomevalonate kinase; MVA-5-pp, mevalonate-5-diphosphate; PMD, diphosphomevalonate decarboxylase; GA-3-p, D-Glyceraldehyde 3-phosphate; DXS, 1-deoxy-D-xylulose 5-phosphate synthase; DXP, 1-deoxy-D-xylulose-5-phosphate; DXR, 1-deoxy-D-xylulose 5-phosphate reductoisomerase; MEP, 2-C-methyl-D-erythritol 4-phosphate; CMS, 2-C-methyl-D-erythritol-4-phosphate cytidylyltransferase; CME, 2-C-methyl-D-erythritol-4-phosphate; CMK, 4-(cytidine 5’-diphospho)-2-C-methyl-D-erythritol kinase; PCME, 4-(cytidine 5’-diphospho)-2-C-methyl-D-erythritol; MCS, 2-C-methyl-D-erythritol 2,4-cyclodiphosphate synthase; CMEC, 2-C-methyl-D-erythritol 2,4-cyclodiphosphate; HDS, 4-hydroxy-3-methylbut-2-enyl diphosphate synthase; HMED, 4-hydroxy-3-methyl-but-2-en-1-yl diphosphate; HDR, 4-hydroxy-3-methylbut-2-enyl diphosphate reductase; IPPI, isopentenyl diphosphate isomerase; GPPS, geranyl pyrophosphate synthase; FPS, farnesyl pyrophosphate synthase; GGPS, geranylgeranyl pyrophosphate synthase; CPT, cis-prenyltransferase; REF, rubber elongation factor; SRPP, small rubber particle protein; CPTL, CPT-like

Previously, a total of 85 genes were identified as rubber biosynthesis genes, including 18 involved in the MVA pathway, 22 in MEP, 15 in APP initiator biosynthesis and 30 in rubber particle-associated rubber elongation [3, 6, 26]. Forty-nine genes among these rubber biosynthesis genes were identified as DEGs in the four comparisons. A simple mode diagram of the rubber biosynthesis pathway was drawn, and the FPKM values of the rubber biosynthesis genes in four latex samples are shown in the form of a heatmap (Fig. 3) and listed in Table S2. In Reyan73397y1 as compared with RRIM600y1, 2 DEGs (HMGR4 and SRPP6) were upregulated, while 3 DEGs (REF4, CPT9 and DXR1) were downregulated. In addition to HMGR4, the rest of the MVA pathway genes showed slightly higher expression levels in Reyan73397y1 than in RRIM600y1, although they did not reach the DEG thresholds. A total of 27 DEGs were upregulated and 3 DEGs (HMGR1, MCS2 and GGPS4) were downregulated in RRIM600y30 compared with Reyan73397y30, among which 13 genes in the MVA pathway and 9 genes in the MEP pathway were upregulated (Table S2 and Fig. 3). To initiate rubber biosynthesis, IPP was first converted to its highly electrophilic isomer, DMAPP, which is catalyzed by the isopentenyl diphosphate isomerase (IPPI) gene. Our results showed that both IPPI1 and IPPI2 were present at relatively low levels in latex. IPPI1 was comparably expressed among the four latex samples, while IPPI2 showed higher expression levels in latex from 1-year-old trees than in that from 30-year-old trees (Table S2 and Fig. 3). A group of *trans*-prenyltransferases, including GGPS, FPPS and GGPPS, catalyze the biosynthesis of APPs, which can serve as rubber molecule initiators and also the precursors of non-rubber isoprenoids. The expression levels of these *trans*-prenyltransferase genes were downregulated in Reyan73397y30 compared with those in RRIM600y30 but displayed no significant difference between those in RRIM600y1 and Reyan73397y1. In addition, RRIM600y30 latex accumulated significantly higher levels of CPT and CPTL transcripts than Reyan73397y30 latex.

**Fig. 4:**
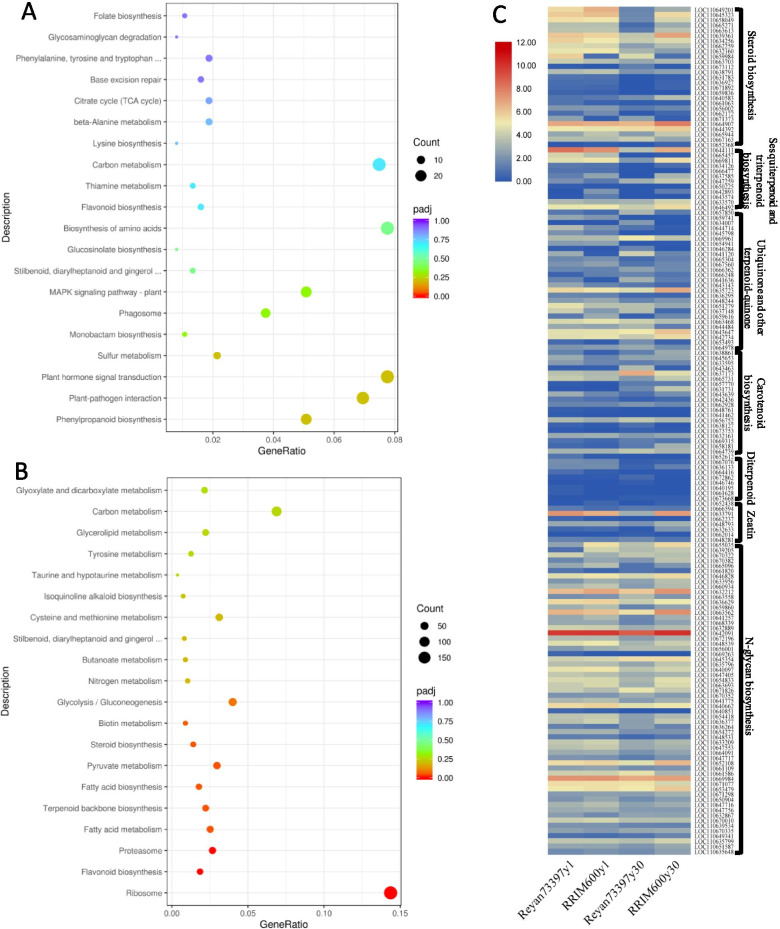
Enriched pathways in comparisons of latex from same-aged Reyan7-33–97 and RRIM600 trees. **a** and **b** indicate the enriched KEGG pathways in comparisons of 6y1vs7y1 and 6y30vs7y30, respectively. **c** Expression profiles of the differentially expressed genes in those enriched downstream non-rubber isoprenoid pathways

KEGG pathway enrichment analysis and gene set enrichment analysis (GSEA) were performed to further investigate the expression levels of genes in pathways related to rubber biosynthesis. Enriched KEGG pathways in comparisons of 6y1vs7y1 and 6y30vs7y30 are respectively shown in Figs. 4a and b, and the enriched gene sets related to isoprenoid pathways from GSEA are listed in Table S3. Pyruvate metabolism (pop00620) and glycolysis/gluconeogenesis (pop00010) pathways, which are the pathways producing pyruvate and acetyl-CoA precursors for IPP biosynthesis, were enriched in RRIM600y30 compared with those in Reyan73397y30. The terpenoid backbone biosynthesis pathway (pop00900) was significantly enriched in RRIM600y30, which contributes to higher levels of rubber biosynthetic enzymes. In Reyan73397y30 as compared with RRIM600y30, the ribosome, proteasome and flavonoid pathways were significantly upregulated, while fatty acid biosynthesis and metabolism genes were downregulated (Fig. 4b). In the comparison of Reyan73397y1 vs. RRIM600y1, non-rubber isoprenoid pathways, including carotenoid biosynthesis (pop00906), zeatin biosynthesis (pop00908), ubiquinone and other terpenoid quinine biosynthesis (pop00130) and diterpenoid biosynthesis (pop00904), were enriched in the latex of Reyan73397y1, and the N-glycan biosynthesis (pop00510) pathway was enriched in that of RRIM600y1 (Table S3). In latex from 30-year-old trees, the non-rubber isoprenoid branches, including sesquiterpenoid and triterpenoid biosynthesis (pop00909), steroid biosynthesis (pop00100), zeatin biosynthesis (pop00908) and N-glycan biosynthesis (pop00510), showed higher expression levels in RRIM600 latex than in Reyan7-33–97 latex (Table S3). To clearly show the expression patterns of the DEGs in these enriched non-rubber isoprenoid pathways, their FPKM values are shown in Table S4 and Fig. 4c.

### Expression levels of genes encoding rubber particle associated proteins

REF and SRPP are the major proteins associated with rubber particles. Comparative transcriptome analysis in our present study showed that the REF and SRPP genes are differentially expressed among these latex samples. As shown in Fig. 3 and Table S2, the majority of REF genes showed higher expression levels in latex from 30-year-old trees than in that from 1-year-old trees, except for REF5 and REF8, which showed lower expression levels in Reyan73397y30. Additionally, latex from 30-year-old trees displayed a higher abundance of transcripts for 4 SRPP isoforms (SRPP1, SRPP2, SRPP3 and SRPP4), while expressed the rest of SRPP genes at lower levels than those in 1-year-old trees (Fig. 3 and Table S2).

**Fig. 5:**
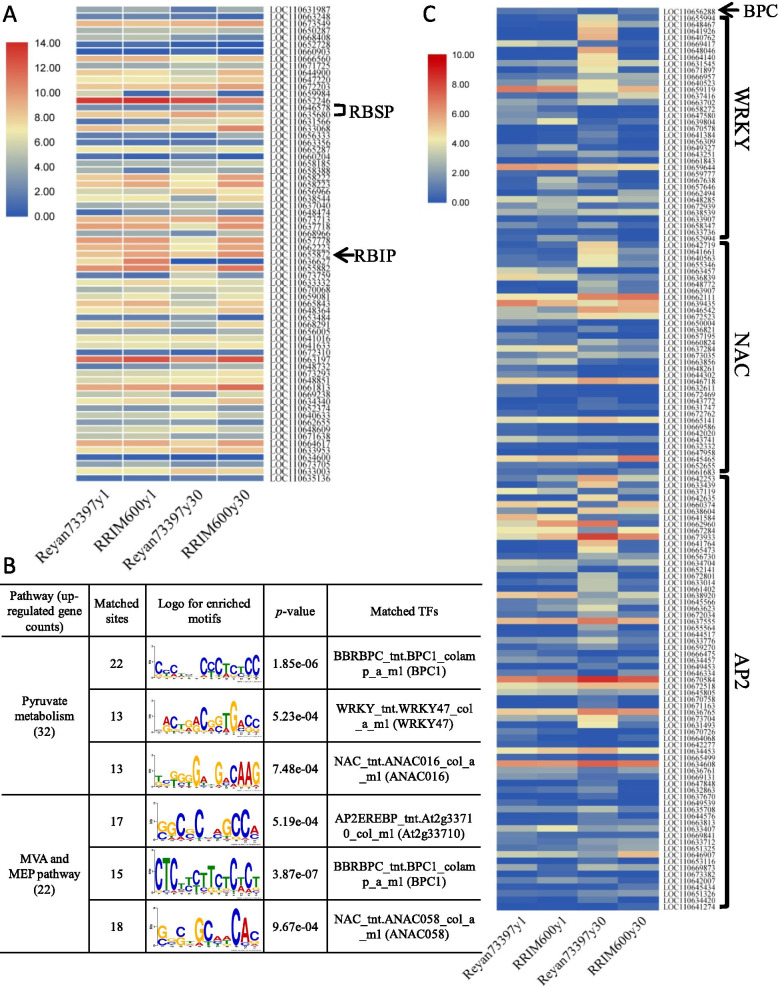
Analysis of differentially expressed rubber particle associated protein genes and IPP biosynthesis-related transcription factors (TFs). **a** Expression profiles of the differentially expressed rubber particle associated proteins. Two rubber biosynthesis stimulator protein (RBSP) genes and one rubber biosynthesis inhibitor protein (RBIP) gene are indicated. **b** The top 3 enriched motifs in the promoter regions of upregulated genes in pyruvate metabolism, MVA and MEP pathways in RRIM600y30 latex and the significantly matched TF families. **c** Expression profiles of the differentially expressed TFs belonging to the BPC, WRKY, NAC and AP2 families are shown in the form of a heatmap. The sample names are shown at the bottom

In addition to REF/SRPP genes, we identified 136 transcripts encoding rubber particle associated proteins based on the previously published proteome data of rubber particles [27]. Among these genes, 68 were differentially expressed among the four latex samples, and their expression levels are listed in Table S5 and Fig. 5a. Several genes that have been previously identified as rubber biosynthesis regulators were shown, including rubber biosynthesis stimulator protein (RBSP) and rubber biosynthesis inhibitor protein (RBIP). In trees of the same age, latex from RRIM600 showed higher expression levels of RP proteins and smaller RPs than latex from Reyan7-33–97 trees.

### Analysis of differentially expressed TFs that are potentially related to IPP biosynthesis

A higher supply of IPP precursors is essential for increasing the yield and MW of rubber. However, current knowledge of the transcriptional regulation of IPP-generating pathways in *Hevea* is very limited. In our present study, a total of 3077 genes with detectable expression levels in *Hevea* latex were identified as transcriptional regulators. Among these genes, 466 were differentially expressed among the four latex samples, and their FPKM values are listed in Table S6. IPP is biosynthesized using sucrose as the raw material, which is first metabolized into pyruvate and then into acetyl-CoA [2, 35]. We thus investigated the TFs that are potentially involved in the regulation of pyruvate metabolism and IPP biosynthetic pathways. Based on the above findings, 32 genes in pyruvate metabolism and 22 genes in the MVA and MEP pathways showed significantly higher expression levels in RRIM600y30 than in Reyan73397y30. Therefore, the *cis*-regulatory elements on the promoters (− 1 to − 1000 bp from the transcription start site) of these upregulated genes were searched for enriched motifs using the MEME suite [36]. The top three statistically significant motifs (depending on the *E*-value) and their corresponding TF families are shown in Fig. 5b. The results showed that the recognition sites of four TF families, including BASIC PENTACYSTEINE (BPC), WRKY, NAC and AP2, were significantly enriched in the promoters of those upregulated DEGs in the pyruvate metabolism, MVA and MEP pathways in RRIM600y30. These TFs are thought to be involved in the regulation of IPP biosynthesis. As shown in Fig. 5c, among the differentially expressed TFs, we identified only one BPC gene (LOC110656288) whose expression level was relatively low in *Hevea* latex and appeared to be positively correlated with the expression levels of IPP biosynthetic pathways. Interestingly, a large group of WRKY, NAC and AP2 genes showed significantly higher expression levels in Reyan73397y30 than in RRIM600y30, revealing their potential negative roles in the regulation of IPP production.

## Discussion

Breeding *Hevea brasiliensis* clones with higher rubber quality is our long-term goal. Thus, a comprehensive understanding of the molecular weight regulation during rubber biosynthesis is required to enlighten the effective molecular modification to predictably improve rubber quality in *Hevea*. According to previous findings [23, 25], the MW of rubber has clonal differences and close correlations with the age of *Hevea* trees, which provides a direction for studying the regulatory mechanism of rubber MW. We therefore determined the number-average molecular weight (MN), weight-average molecular weight (MW) and molecular weight distributions (MWD) of latex from 1-year virgin trees and 30-year-old regularly tapped trees of the *Hevea* clones Reyan7-33–97 and RRIM600. GPC results indicated that the average MW of *Hevea* latex varied between the Reyan7-33–97 and RRIM600 clones and increased with tree age (Table 1). The Reyan73397y1 latex displayed a slightly higher MW than that of RRIM600y1, whereas RRIM600y30 latex had a significantly higher MW than that of Reyan73397y30 in the same trial (Table 1). These results revealed the distinct regulations of latex MW among Reyan7-33–97 and RRIM600 trees and provided a foundation for follow-up studies on the regulatory mechanism of rubber MW.

Rubber biosynthesis takes place on the surface of rubber particles, which are endoplasmic reticulum-derived organelles comprising a hydrophobic polyisoprene core and a surrounding monolayer membrane of lipids and proteins [18]. Correlations between MW and RP size have been extensively investigated. Tarachiwin et al. [17] reported that the size of RPs is negatively correlated with latex molecular weight. Smaller RPs tend to have rubber with a higher average molecular weight in *Hevea* trees. In our present study, both the LLS and TEM results showed that the average RP sizes were smaller in RRIM600 trees than in Reyan7-33–97 trees of the same age. Our results indicated that the latex MW is not directly correlated with the average particle size among these four latex samples. As a recent study showed that the particle size of RPs is not a determinant of the molecular size of NR [19], further investigations are required to determine whether the size variations of RPs play a role in the formation of clonal and age-related differences in latex MW.

The differences in latex and RP size could be associated with rubber particle membrane proteins. Small RPs comprise a higher abundance of proteins and exhibit higher rubber transferase (RT-ase) enzymatic activities than larger RPs [19, 20]. As the most abundant proteins on rubber particles, REFs and SRPPs are homologous proteins with distinct properties. SRPPs show less affinity for membranes and cover lipid headgroups without disturbing membrane integrity, whereas REFs show higher membrane affinity, insert into membranes and perturb membrane integrity [37]. Yamashita et al. [19] showed that an SRPP isoform appeared predominantly in the smaller RP fractions, while an isoform of REF was detected predominantly in the larger RP fractions. Transcriptome data showed that, compared with the latex from 1-year-old trees, latex from 30-year-old trees displayed a higher abundance of transcripts for REF and 4 SRPP isoforms (SRPP1, SRPP2, SRPP3 and SRPP4), while expressed the rest of SRPP genes at lower levels. In trees of the same age, both the REF and SRPP genes were highly expressed in RRIM600 latex compared with those in Reyan7-33–97 latex (Fig. 3 and Table S2). These findings suggest the potential involvement of those differentially expressed REF and SRPP proteins in the significant variations in the size and morphology of RPs among the four latex samples. It has been previously suggested that the REF gene expression pattern has a positive correlation with latex yield [38]. Our present study showed inconsistent results: in both 1- and 30-year-old *Hevea* trees, the abundance of REF transcripts was higher in RRIM600 latex than in latex from the high-yielding clone Reyan7-33–97. Similarly, the expression levels of REF isoforms were higher in RRIM600 than in higher-yielding clones PB350 and RRIM901 [33]. Thus, the roles of REF/SRPP in rubber biosynthesis require further investigation.

In addition to REF/SRPP proteins, our results revealed that the overall transcript abundances of RP-associated proteins appeared to be negatively correlated with the average RP size (Fig. 1b, Fig. 5a and Table S5). Additionally, microscopic analysis of rubber particles using TEM showed that some rubber particles in Reyan7-33–97 trees aggregated, while those in RRIM600 remained solitary (Fig. 1). According to Dai et al. [39], RP proteins enable negatively charged particles to be uniformly dispersed in *Hevea* latex. We propose that in trees of the same age, the smaller rubber particle size in RRIM600 latex is likely attributed to the higher abundance of rubber particle proteins, which confers enhanced electrostatic repulsion that prevents rubber particles from aggregating into larger particles.

Among these RP-localized proteins, a patatin-like protein (LOC110655872) was previously identified as a rubber biosynthesis inhibitor protein (RBIP) [24]. LOC110655872 was found to have significantly higher expression levels in RRIM600 latex than in latex from high-yielding Reyan7-33–97 trees (Table S5 and Fig. 5a). Eukaryotic initiation factor 5A (eIF-5A), a factor involved in the formation of the first peptide during protein synthesis, was previously identified as a rubber biosynthesis stimulator protein (RBSP) [40]. Among the two differentially expressed eIF genes, eIF-5A (LOC110635680) showed twofold higher expression levels in latex of the high-yielding clone Reyan7-33–97 than that of RRIM600 (Table S5 and Fig. 5a). Our results suggest the potential involvement of these differentially expressed rubber biosynthesis regulators in the different yield traits of RRIM600 and Reyan7-33–97 trees.

Expression levels of rubber biosynthesis-related genes were investigated, and the results showed that in 1-year-old trees, Reyan7-33–97 latex displayed slightly higher expression levels of MVA pathway genes than RRIM600 latex, suggesting higher levels of IPP biosynthesis in Reyan73397y1 latex than in RRIM600y1 latex. In latex from 30-year-old trees, the glycolysis/gluconeogenesis (pop00010), pyruvate metabolism (pop00620), terpenoid backbone biosynthesis (pop00900), MVA and MEP pathways were higher expressed in RRIM600y30 than in Reyan73397y30 (Table S2, Fig. 4b and Table S3). Altogether, our present study showed interesting results that in 30-year-old regularly tapped trees, the latex from the average-yielding RRIM600 clone appeared to have higher levels of acetyl-CoA and IPP precursors and rubber biosynthetic activities than the latex from the high-yielding Reyan7-33–97 clone. This trend of relative transcriptional activity appears to reflect the observations from a previous comparative study between RRIM600 and the higher-yielding clones PB350 and RRIM901 [33].

Some earlier studies demonstrated the effects of genes regulating various APP biosynthesis on the MW and yield of latex in rubber-producing plants. Overexpression of EuIPPI, which catalyzes the first step in the biosynthesis of all APP initiators, resulted in increased yield and MW of transpolyisoprenes in transgenic *E. ulmoides* [41]. More rubber molecules were synthesized in transgenic *P. argentatum* plants overexpressing the *FPS* and *GGPPS* genes than in wild-type plants, but these molecules were of lower molecular weight [42]. Similarly, our another ongoing study shows that *Hevea* trees overexpressing an exogenous *FPS* gene exhibited increased expressions of both two *IPPI* genes and a slightly decreased average MW of latex, and their yield traits require further investigation (unpublished data). As previously observed in in vitro experiments, under limited IPP concentrations, increasing initiators cause a higher yield of latex with a lower MW [7, 16]. These findings revealed the significant roles of those processes that produce and consume IPP and APPs in regulating the MW and yield of latex.

Further comparative analyses of the transcriptome profiles provided insights into the mechanisms underlying different latex MW and yield traits of the regularly tapped trees of RRIM600 and Reyan7-33–97 clones. Our results showed that in latex from *Hevea* trees of the same age, IPPI genes were comparably expressed (Fig. 3 and Table S2), which suggests that the efficiencies for the interconversion of IPP to DMAPP are comparable among RRIM600 and Reyan7-33–97 latex. It is worth noting that those gene sets of downstream non-rubber isoprenoid pathways were differentially expressed among latex from same-aged RRIM600 and Reyan7-33–97 trees (Table S3), which consume APP initiators for producing functionally and structurally diverse non-rubber isoprenoid products. In the comparison Reyan73397y1 vs RRIM600y1, carotenoid biosynthesis (pop00906), zeatin biosynthesis (pop00908), ubiquinone and other terpenoid quinine biosynthesis (pop00130) and diterpenoid biosynthesis (pop00904) pathways were significantly enriched in latex from Reyan73397y1, and the N-glycan biosynthesis (pop00510) pathway was enriched in that from RRIM600y1 (Table S3). In latex from 30-year-old trees, the non-rubber isoprenoid branches, including sesquiterpenoid and triterpenoid biosynthesis (pop00909), steroid biosynthesis (pop00100), zeatin biosynthesis (pop00908) and N-glycan biosynthesis (pop00510), showed higher expression levels in RRIM600 latex than in Reyan7-33–97 latex (Table S3). Therefore, under limited DMAPP supply, RRIM600y30 latex appeared to have less APP initiator flux in the rubber biosynthesis branch, which, together with a greater supply of IPP, resulted in fewer rubber molecules with increased chain lengths. Additionally, in 1-year-old trees, compared with RRIM600y1 latex, the higher-MW Reyan73397 latex was supposed to have higher levels of IPP and lower levels of APPs, which resulted from the slightly higher expressed MVA pathway and the enriched non-rubber isoprenoid pathways, respectively (Fig. 4c and Table S3).

**Fig. 6:**
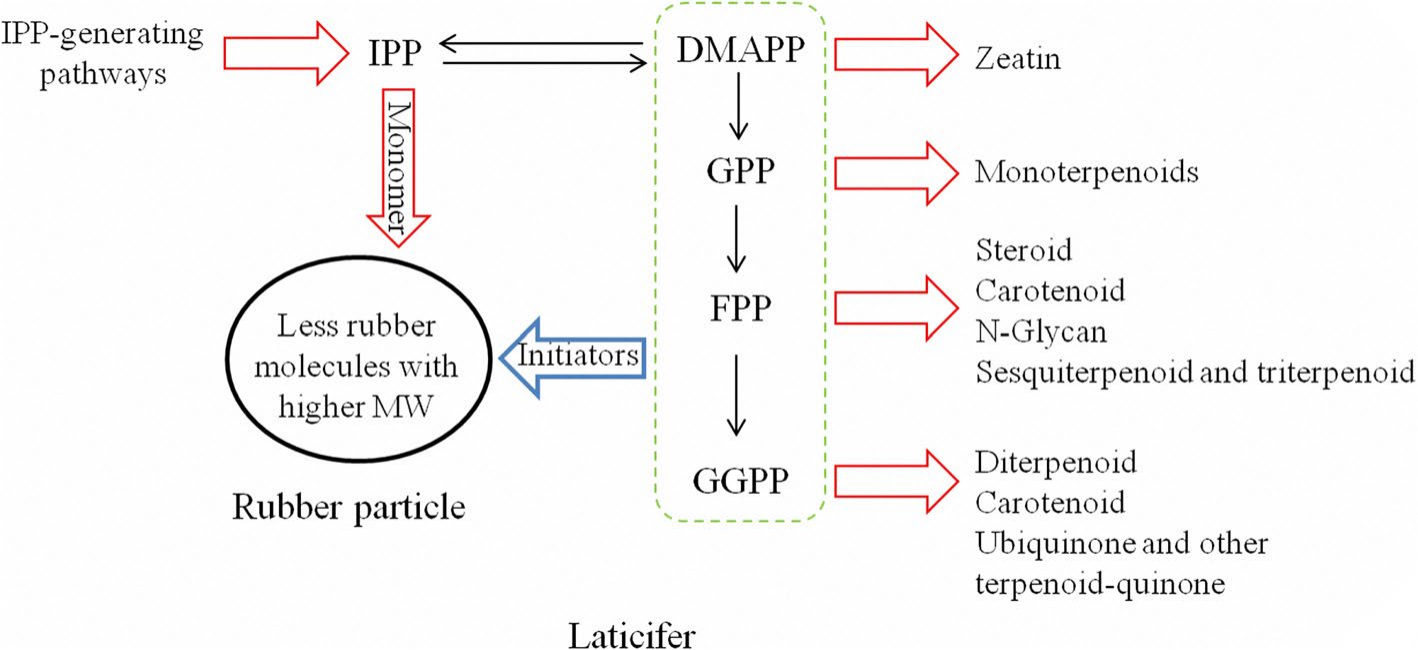
A schematic diagram showing the effects of the differentially expressed IPP-generating pathways and the non-rubber isoprenoid pathways on rubber molecular weight. The red arrows indicate the upregulated pathways and monomers, whereas the blue arrow indicates the downregulated initiators

Based on the above findings, we propose that the variations in latex MW among RRIM600 and Reyan7-33–97 trees of the same age likely resulted from different expression patterns of IPP-generating pathways and the pathways utilizing APPs for the production of rubber and non-rubber isoprenoids (Fig. 6). Rubber MW increases with IPP concentration when APPs are limiting. Under limited supply of DMAPP and APPs, upregulated non-rubber isoprenoid pathways reduce APP flux in rubber biosynthesis branch, resulting in increased rubber MW and decreased latex yield, while downregulating non-rubber pathways has the opposite effects. Further biochemical studies are required to quantify the pathway intermediates IPP, DMAPP and APP initiators, and the downstream rubber and non-rubber products. And also need to compare the affinity of RT-ases and non-rubber APP-requiring enzymes for APPs to understand how rubber and non-rubber branches compete for APP initiators or how this is regulated.

However, comparisons of latex from 1-year virgin trees and 30-year-old regularly tapped trees suggested that there are other factors affecting the chain-length of rubber molecules. Although higher expressed the MVA and MEP pathways, as well as the downstream non-rubber isoprenoid pathways, the latex from 1-year-old virgin trees displayed significantly lower molecular weights than those from 30-year-old regularly tapped trees (Table 1, Fig. 3 and Table S3). This is probably due to the fact that, in rapidly growing young trees, IPP is utilized to produce a large group of compounds essential for plant growth processes, and rubber is made only when IPP is in excess of their requirements. As previously reviewed by Cherian et al. [3], all RT-ases investigated to date have high Km ^IPP^’s, preventing rubber from polymerizing IPP in competition with essential IPP-requiring enzymes.

On the basis of the knowledge that a higher supply of acetyl-CoA and IPP precursors in latex is essential for increasing the yield and MW of rubber, we therefore investigated the TFs that are potentially involved in the regulation of acetyl-CoA and IPP biosynthetic pathways. We identified 139 differentially expressed TFs belonging to four families (BPC, WRKY, NAC and AP2); their recognition sites are significantly enriched in the promoters of the upregulated DEGs in pyruvate metabolism, MVA and MEP pathways in RRIM600y30. Shanks et al. [43] suggested that BPCs are involved in the response to cytokinin and play diverse roles in plant growth and development. Currently, there is no information on how BPCs regulate rubber biosynthesis in *Hevea* trees. A differentially expressed BPC gene (LOC110656288), although present at a very low level, appeared to be positively correlated with the expression levels of IPP biosynthetic pathways, revealing its potential roles in promoting rubber biosynthesis in *Hevea*. A group of NAC transcription factors were previously identified as regulators of the cold stress response [44]. Cao et al. [45] reported that *HbNAC1* (LOC110641661) is involved in dehydration-induced laticifer differentiation and latex biosynthesis. Our results showed that the expression of *HbNAC1* in latex increases with tree-age, and was significantly higher in Reyan73397y30 than in RRIM600y30, which is inconsistent with the expression of IPP-producing pathways but coincides with the latex yield traits of these two *Hevea* clones. It has been previously demonstrated that *HbWRKY1* (LOC110661399) and *HbWRKY14* (LOC110662072) suppress the expression of SRPP and behave as negative regulators of NR biosynthesis [3, 46, 47]. Although *HbWRKY1* and *HbWRKY14* were not differentially expressed among these four latex samples, our results showed that a large group of other WRKYs had significantly higher expression levels in Reyan73397y30 than in RRIM600y30, which may account for the lower abundance of SRPP transcripts in Reyan73397y30. AP2 transcription factors are essentially involved in the ethylene signalling pathway, which regulates rubber biosynthesis in *Hevea* [2]. Makita et al. [33] confirmed the potential binding elements of AP2 in the promoters of CPT genes in *Hevea* trees. A total of 67 AP2 genes were differentially expressed among the four latex samples, 20 and 8 of which were up- and downregulated, respectively, in Reyan73397y30 compared with that in RRIM600y30 (Table S6), indicating that these AP2 genes are likely under different transcriptional regulations among Reyan73397y30 and RRIM600y30 latex. Further studies are required to elucidate the regulatory roles of these differentially expressed TFs in regulating the biosynthesis and MW of rubber in *Hevea* trees.

## Conclusions

In our present study, we comparatively analyzed the MW, rubber particle size and transcriptome profiles of latex from 1-year-old virgin trees and 30-year-old regularly tapped trees of the *Hevea* clones Reyan7-33–97 and RRIM600. Our results showed that the average MW and RP size of latex varied between the Reyan7-33–97 and RRIM600 clones and increased with tree age. Transcriptome analysis suggests that the smaller average particle size in RRIM600 latex is likely attributed to higher expression levels of RP-associated proteins, and the MW variations among Reyan7-33–97 and RRIM600 latex appeared to have resulted from different expression patterns of those pathways supplying and utilizing IPP and APP initiators for rubber and non-rubber isoprenoids (Fig. 6). In addition, a group of transcription factors that may play roles in IPP biosynthesis were identified. Taken together, our results suggested that precise modulation of the levels and ratios of IPP and APPs in rubber biosynthesis, by fine-tuning the expression of IPP-generating pathways and the pathways consuming APPs for non-rubber isoprenoids, may be a viable engineering strategy for increasing the MW and yield of latex.

## Methods

### Plant Materials

The *Hevea* trees used in present study were obtained by grafting Reyan7-33–97 and RRIM600 scions onto seed-seedling rootstocks and grown at “National Tropical Plants Germplasm Resource Center” and an experimental plantation in Hainan Province, China. Fresh latex and bark samples were collected from 1-year-old virgin trees and 30-year-old regularly tapped trees of clones Reyan7-33–97 and RRIM600, and designated Reyan73397y1, RRIM600y1, Reyan73397y30 and RRIM600y30, respectively. Three individual trees per clone at each age were sampled. Necessary permissions to collect these samples were provided by CATAS (Chinese Academy of Tropical Agricultural Sciences, Danzhou, Hainan, China). The bark samples were used to study the morphologies of rubber particles within laticifers, and the latex samples were used for the analysis of MW, MWD, transcriptome and latex particle distributions.

### Determination of the molecular weight and particle size of latex

The molecular weight distributions of the latex samples were measured by gel permeation chromatography (GPC). For GPC measurement, dried latex samples were dissolved in tetrahydrofuran (THF) at a concentration of 5 mg/ml. A Waters GPC system consisted of two columns with exclusion limits of 2.0 × 10^7^ and 5.0 × 10^4^, and a differential refractive index detector was applied to the samples. THF was used as the mobile phase at a flow rate of 0.5 ml/min. Commercially obtained polyisoprene standards of known molecular weight were used to calibrate the columns. The measurement temperature was set at 35 °C, and the injection volume was 10 μl. MW information of each latex sample was collected and analyzed using Agilent (Santa Clara, USA) GPC/SEC Software (version 1.2). The mean and standard deviation of latex MW were calculated from three biological replicates.

The sizes and distributions of RPs in each latex sample were determined by an LA-960 (HORIBA Scientific, Japan) Light Scattering Particle Size Distribution Analyzer at 30 °C with a 90° determination angle.

### Bark anatomy and transmission electron microscopy

Ultrathin cross sectioning of bark samples was carried out as described previously [48]. The morphology of rubber particles within the bark laticifers was examined using a HT7700 TEM system (Hitachi, Japan) at 80 kV with 4000 magnification.

### Latex RNA isolation and transcriptome sequencing

The latex samples were frozen immediately by dipping in liquid nitrogen and ground into fine powder. Total RNA was extracted according to the manufacturer’s protocol using TRIzol reagent (Invitrogen) and with an additional DNase I to remove any traces of genomic DNA. The purified RNA was quantified using a NanoDrop 2000 spectrophotometer (Thermo Fisher Scientific, USA). Whole transcriptome cDNA library construction and Illumina sequencing were performed by Novogene Corporation (Tianjin, China). For each latex sample, three biological replicates were sequenced and analyzed.

### Sequence data assembly and functional annotation

Raw data in FASTQ format were subjected to stringent filtering conditions for the removal of adaptor sequences, low-quality reads and reads with ambiguous bases. All clean reads from latex samples were assembled using Trinity 2.0 [49] and mapped to the previously published reference genome of *Hevea brasiliensis* (NCBI bioproject: PRJNA394253) [26]. Functional annotations of assembled unigenes were performed by searching sequences against the following public databases: NCBI non-redundant protein sequences (Nr), NCBI non-redundant nucleotide sequences (Nt), SwissProt protein sequence database, Clusters of Orthologous Groups of proteins (COG), Kyoto Encyclopedia of Genes and Genomes database (KEGG), and Gene Ontology (GO) with blastx. The expression levels of the assembled unigenes were calculated by the fragments per kilobase per million fragments method [50]. The DEGs between the latex samples were assessed with *p*-value < 0.005 and |log2FoldChange|> 1 thresholds [51]. Heatmaps and Venn diagrams were constructed using TBtools to analyze the expressions and relationships of DEGs among different comparisons [52]. GO enrichment analysis of the DEGs was performed using the GOseq R package based on the Wallenius non-central hyper-geometric distribution [53]. KEGG pathway enrichment and Gene Set Enrichment Analysis (GSEA) of the DEGs were performed using KOBAS and GSEA software, respectively [54, 55].

### qRT-PCR analysis

To validate the data from transcriptome analysis, qRT-PCR was performed. The expression patterns of 17 genes encoding key rubber biosynthetic enzymes and regulators in four latex samples were determined using the *HbRH8* (LOC110669478) gene as the internal control. Total RNA was reverse transcribed into cDNA using an Easyscript cDNA synthesis kit with gDNA remover (Transgen, China). Diluted cDNA was amplified with specific primers (listed in Table S7) and reacted with TransStart Tip Green qPCR SuperMix (Transgen, China) on a CFX96 Real-Time PCR System (Bio-Rad) under the following conditions: 45 cycles of 94 °C for 5 s, 56 °C for 15 s and 72 °C for 10 s. The expression levels of each gene were calculated using the formula 2^−△△Ct^. For each latex sample, three biological replicates were performed. The correlations between transcriptome analysis and qRT-PCR data were calculated using the relative expression levels of these 17 genes.

### Identification and categorization of transcriptional regulators in rubber latex

The expressed transcripts in latex samples were submitted to the online iTAK analysis tool (http://itak.feilab.net/cgi-bin/itak/index.cgi) for identification and automatic classification of transcription factors [56].

### Promoter motif analysis

To identify the potential *cis*-regulatory elements and transcription factors involved in latex molecular weight regulation, we studied the promoters of those upregulated genes in IPP-generating pathways in latex samples from 30-year-old trees of RRIM600. The 1000 bp sequences upstream of the transcription start site of these genes were extracted for motif discovery. The MEME suite was used in classic mode to identify enriched motifs [36], with the following parameters: maximum number of motifs = 12, minimum motif width = 6, maximum motif width = 14 and background model set to 1st order. Using the TomTom tool from the MEME suite with default parameters, the top three statistically significant (low *E*-value) motifs for each gene set were searched against the *Arabidopsis* DAP motif database to identify potential transcription factors (TFs) capable of recognizing those enriched motifs. The expression levels of these TF genes in four latex samples were further analyzed.

## Supplementary Information

Additional file 1: **Table S1**. Sequencing statistical summary of latex transcriptomes.

Additional file 2: **Table S2**. Expression levels (FPKM) of rubber biosynthetic genes in four H. brasiliensis latex samples.

Additional file 3: **Table S3**. The differentially enriched gene sets related to isoprenoid pathways among latex samples.

Additional file 4: **Table S4**. Expression levels (FPKM) of differentially expressed non-rubber isoprenoid pathway genes in four H. brasiliensis latex samples.

Additional file 5: **Table S5**. Differentially expressed rubber particle associated proteins among four H. brasiliensis latex samples.

Additional file 6: **Table S6**. Classifications and the FPKM values of the differentially expressed transcription factors in latex samples.

Additional file 7: **Table S7**. Specific primers used for quantivtative real-time PCR of 17 key genes.

Additional file 8: **Fig. S1**. Hierarchical cluster analysis indicated that the expression patterns of DEGs obtained from the transcriptome sequencing analysis could be divided into 4 sub-clusters.

Additional file 9: **Fig. S2**. Correlation analyses of the RNA-Seq (FPKM) and qRT-PCR results. The values of log2 of expression levels fold change in qRT-PCR (x-axis) were plotted against the values of log2 of FPKM fold changes in transcriptome data (y-axis) for the 17 selected genes in four latex samples.

## Data Availability

All raw sequence data generated in this study have been deposited at National Center for Biotechnology Information (NCBI) in the Short Read Archive database under the bioproject accession number: PRJNA688380.

## Abbreviations

MW: Weight-average molecular weight
MN: Number-average molecular weight
MWD: Molecular weight distribution
RP: Rubber particle
TEM: Transmission electron microscope
LLS: Laser light scattering
FPKM: Fragments per kilobase per million fragments
MVA: Mevalonate
MEP: 2-C-Methyl-D-erythritol 4-phosphate
ACAT: Acetyl-CoA acetyltransferase
HMGS: 3-Hydroxy-3-methylglutaryl-CoA synthase
HMG-CoA: Hydroxymethylglutaryl coenzyme A
HMGR: 3-Hydroxyl-3-methylglutaryl-CoA reductase
MVK: Mevalonate kinase
MVA-5-p: Mevalonate-5-phosphate
MVD: Phosphomevalonate kinase
MVA-5-pp: Mevalonate-5-diphosphate
PMD: Diphosphomevalonate decarboxylase
GA-3-p: D-Glyceraldehyde 3-phosphate
DXS: 1-Deoxy-D-xylulose 5-phosphate synthase
DXP: 1-Deoxy-D-xylulose-5-phosphate
DXR: 1-Deoxy-D-xylulose 5-phosphate reductoisomerase
CMS: 2-C-methyl-D-erythritol 4-phosphate cytidylyltransferase
CME: 2-C-methyl-D-erythritol-4-phosphate
CMK: 4-(Cytidine 5’-diphospho)-2- C-methyl-D-erythritol kinase
PCME: 4-(Cytidine 5’-diphospho)-2-C-methyl-D-erythritol
MCS: 2-C-methyl-D-erythritol 2,4-cyclodiphosphate synthase
CMEC: 2-C-methyl-D-erythritol 2,4-cyclodiphosphate
HDS: 4-Hydroxy-3-methylbut-2-enyl diphosphate synthase
HMED: 4-Hydroxy-3- methyl-but-2-en-1-yl diphosphate
HDR: 4-Hydroxy-3-methylbut-2-enyl diphosphate reductase
IPPI: Isopentenyl diphosphate isomerase
GPPS: Geranyl pyrophosphate synthase
FPS: Farnesyl pyrophosphate synthase
GGPS: Geranylgeranyl pyrophosphate synthase
CPT: *Cis*-Prenyltransferase
CPTL: CPT-like
REF: Rubber elongation factor
SRPP: Small rubber particle protein
