# Supplementary material for: Comparative analysis of latex transcriptomes reveals the potential mechanisms underlying rubber molecular weight variations between the Hevea brasiliensis clones RRIM600 and Reyan7-33–97
Source: BMC Plant Biol. 2021 May 29;21:244. doi: 10.1186/s12870-021-03022-5 (PMC8164328; doi:10.1186/s12870-021-03022-5)
Supplement: Supplementary file 8 — Fig. S1. Hierarchical cluster analysis indicated that the expression patterns of DEGs obtained from the transcriptome sequencing analysis could be divided into 4 sub-clusters. [file 12870_2021_3022_MOESM8_ESM.docx]

Comparative analysis of latex transcriptomes reveals the potential mechanisms underlying the rubber molecular weight variations among *Hevea brasiliensis* clones RRIM600 and Reyan7-33-97

Shichao Xin, Yuwei Hua, Ji Li, Xuemei Dai, Xianfeng Yang, Jinu Udayabhanu, Huasun Huang* and Tiandai Huang*


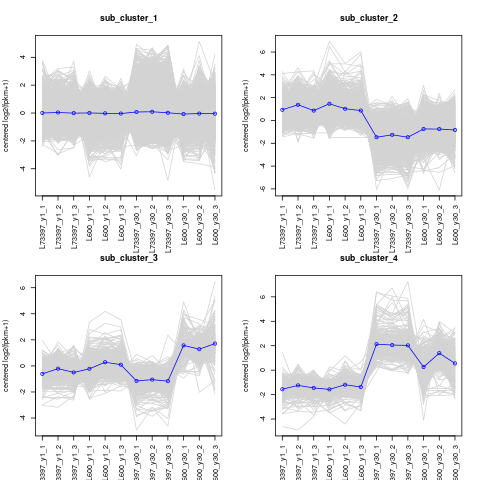


**Fig. S1.** Hierarchical cluster analysis indicated that the expression patterns of DEGs obtained from the transcriptome sequencing analysis could be divided into 4 sub-clusters.
