# Supplementary material for: Comparative analysis of latex transcriptomes reveals the potential mechanisms underlying rubber molecular weight variations between the Hevea brasiliensis clones RRIM600 and Reyan7-33–97
Source: BMC Plant Biol. 2021 May 29;21:244. doi: 10.1186/s12870-021-03022-5 (PMC8164328; doi:10.1186/s12870-021-03022-5)
Supplement: Supplementary file 9 — Fig. S2. Correlation analyses of the RNA-Seq (FPKM) and qRT-PCR results. The values of log2 of expression levels fold change in qRT-PCR (x-axis) were plotted against the values of log2 of FPKM fold changes in transcriptome data (y-axis) for the 17 selected genes in four latex samples. [file 12870_2021_3022_MOESM9_ESM.docx]

Comparative analysis of latex transcriptomes reveals the potential mechanisms underlying the rubber molecular weight variations among *Hevea brasiliensis* clones RRIM600 and Reyan7-33-97

Shichao Xin, Yuwei Hua, Ji Li, Xuemei Dai, Xianfeng Yang, Jinu Udayabhanu, Huasun Huang* and Tiandai Huang*


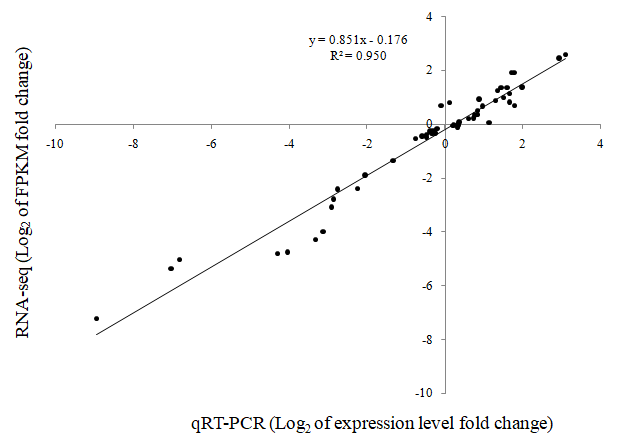


**Fig. S2.** Correlation analyses of the RNA-Seq (FPKM) and qRT-PCR results. The values of log_2_ of expression levels fold change in qRT-PCR (x-axis) were plotted against the values of log_2_ of FPKM fold changes in transcriptome data (y-axis) for the 17 selected genes in four latex samples.
